# Prevalence and severity of anaemia among the Temiar sub-ethnic indigenous Orang Asli communities in Kelantan, Peninsular Malaysia

**DOI:** 10.3389/fpubh.2024.1412496

**Published:** 2024-08-07

**Authors:** Zulkarnain Md Idris, Wathiqah Wahid, Mohd Ikhwan Mukmin Seri Rakna, Nuraffini Ghazali, Noor Wanie Hassan, Siti Nor Azreen Abdul Manap, Ahmad Imran Mohamed, Sriwipa Chuangchaiya, Muhd Rafiq Mohd Kasri

**Affiliations:** ^1^Department of Parasitology and Medical Entomology, Faculty of Medicine, Universiti Kebangsaan Malaysia, Kuala Lumpur, Malaysia; ^2^Gua Musang District Health Office, Kelantan, Malaysia; ^3^Department of Community Health, Faculty of Public Health, Kasetsart University, Sakon Nakhon, Thailand

**Keywords:** anaemia, prevalence, severity, indigenous Orang Asli, Malaysia

## Abstract

**Introduction:**

Anaemia remains a primary concern of public health in developing countries. Indigenous populations are a significant and frequently underreported group at risk for anaemia. This study aimed to assess the prevalence of anaemia and identify its determinants in the Temiar sub-ethnic indigenous Orang Asli (OA) community in Peninsular Malaysia.

**Methodology:**

A community-based cross-sectional study was conducted among 640 indigenous Temiar OA participants from a remote settlement in Gua Musang, Kelantan, Malaysia. Data was collected using face-to-face interviews with a standardised pretested questionnaire and through blood samples collected for haemoglobin (Hb) testing. Anaemia status was determined using the Hb level cut-off established by the World Health Organization (WHO). Descriptive analysis was used to determine the prevalence of anaemia, while multiple logistic regression was used to determine factors associated with anaemia.

**Results:**

The overall anaemia prevalence was 44.7% (286/640), and the prevalence rates of mild, moderate and severe anaemia were 42.7, 50.7 and 6.6%, respectively. Anaemia-specific prevalence varied significantly by age group (*p* < 0.001) and was highest in the ≤5 group for both moderate anaemia (43.4%) and severe (42.1%), followed by the 6–17 age group for mild anaemia (39.3%). The prevalence of anaemia was also highest among students (53.9%), with a significant difference observed between the three anaemia severity classifications (*p* = 0.002). In the multivariate logistic regression, only age groups of 6–17 (adjusted odds ratio [aOR] 0.38, *p* < 0.001), 18–40 (aOR 0.18, *p* < 0.001) and > 40 (aOR 0.25, *p* < 0.001) were significantly associated with the lower odds of anaemia in the population.

**Conclusion:**

This study has highlighted the high prevalence of anaemia among indigenous OA in Peninsular Malaysia and revealed that younger children were positively associated with childhood anaemia. Effective interventions and special attention to this indigenous population need to be implemented to reduce the risk of anaemia.

## 1 Introduction

Anaemia has been identified as a major worldwide public health issue as it affects more than two billion people or represents 24.8% of the world’s population (1). Anaemia is a condition in which the number of red blood cells or the haemoglobin (Hb) concentration within the human body is lower than normal (2, 3). Haemoglobin is required to carry oxygen, and the blood’s capacity to deliver oxygen to the body’s tissues will be reduced if the individual has extremely low or abnormal amounts of red blood cells or insufficient Hb. This condition will lead to general symptoms, including recurrent vagueness, exhaustion, shortness of breath and poor focus (4).

Anaemia has multiple precipitating factors and may result from a multifactorial deficiency of micronutrients (e.g., iron, folic acid, vitamin A and B12), infectious diseases (e.g., malaria and helminth) and inherited disorders (e.g., sickle cell disease and thalassemia) (5, 6). Anaemia affects almost all age groups, from infants to the older adult. However, blood loss from menstruation in women of childbearing age are at a higher risk of acquiring a disease (7). Infants between the ages of 1 and 2 are also at risk for anaemia if they do not consume enough iron in their meals (8). According to the World Health Organization (WHO), 42% of children under five years old and 40% of pregnant women worldwide are estimated to be anaemic (9). Furthermore, people who live in the less developed areas of the country and among hard-to-reach populations are more susceptible to anaemia because of poor quality diet, social–emotional and exposure to parasitic infections (10).

The indigenous population is an important and often unreported group at risk of anaemia (11). Anaemia is a mild concern among indigenous people living in developed countries, but it has become a significant concern in the world’s poorest regions (11). Indigenous communities who are minorities often experience substantial and persistent differences in the socioeconomic determinants of health, such as access to healthcare services, education, work, housing and food security (12). This is related to the causes of anaemia, which include a lack of exercise, unfavourable environmental conditions and an association with infectious diseases like malaria and intestinal parasites (5, 6).

In Malaysia, the indigenous communities known as Orang Asli (OA) represent 0.6% of the total population of Malaysians (13). The OA in Peninsular Malaysia is divided into three main lines (i.e., Negrito, Senoi and Proto-Malay). The Senoi lineage is the largest ethnical group, constituting 55% of the total OA population, with a large majority from the Temiar sub-ethnic (Malaysia Department of Orang Asli). Roughly 37% of 869 OA villages throughout the country are still located in remote and forested areas (14). Poverty and remote settlements have previously been reported to contribute to numerous health problems related to malnourishment and the high prevalence of infectious diseases in these communities (15–26). Although several studies have assessed the nutritional status and metabolic syndrome among the OA in Malaysia, there is a lack of information on anaemia status among this population, especially among the Temiar sub-ethnic indigenous OA communities. A recent study conducted among the Negrito OA from inland jungle villages and resettlement at town peripheries reported an overall prevalence of anaemia among children and adolescents was 68.4% (23). Previous studies have also shown that the contributing factors of anaemia among the indigenous OA population in Malaysia are largely due to poor dietary intake of iron, recurrent infections and low socioeconomic status (27, 28).

This study aimed to determine the prevalence and associated risk factors of anaemia among Temiar sub-ethnic indigenous OA communities in Peninsular Malaysia. Results from this study would be useful for informing national prevention strategies and could be used by several already existing national agencies dedicated to improving morbidity and mortality among indigenous peoples.

## 2 Methods

### 2.1 Ethics statement

This study was conducted in accordance with the Declaration of Helsinki and was approved by the Medical Ethics Committee of the National University of Malaysia (Reference No. UKM PPI/111/8/JEP-2019-148) and the Department of Orang Asli Development, Ministry of Rural and Regional Development Malaysia. Participants were sensitised to the study objectives and procedures by the local health district personnel for the study participation.

### 2.2 Study area and population

**Figure 1:**
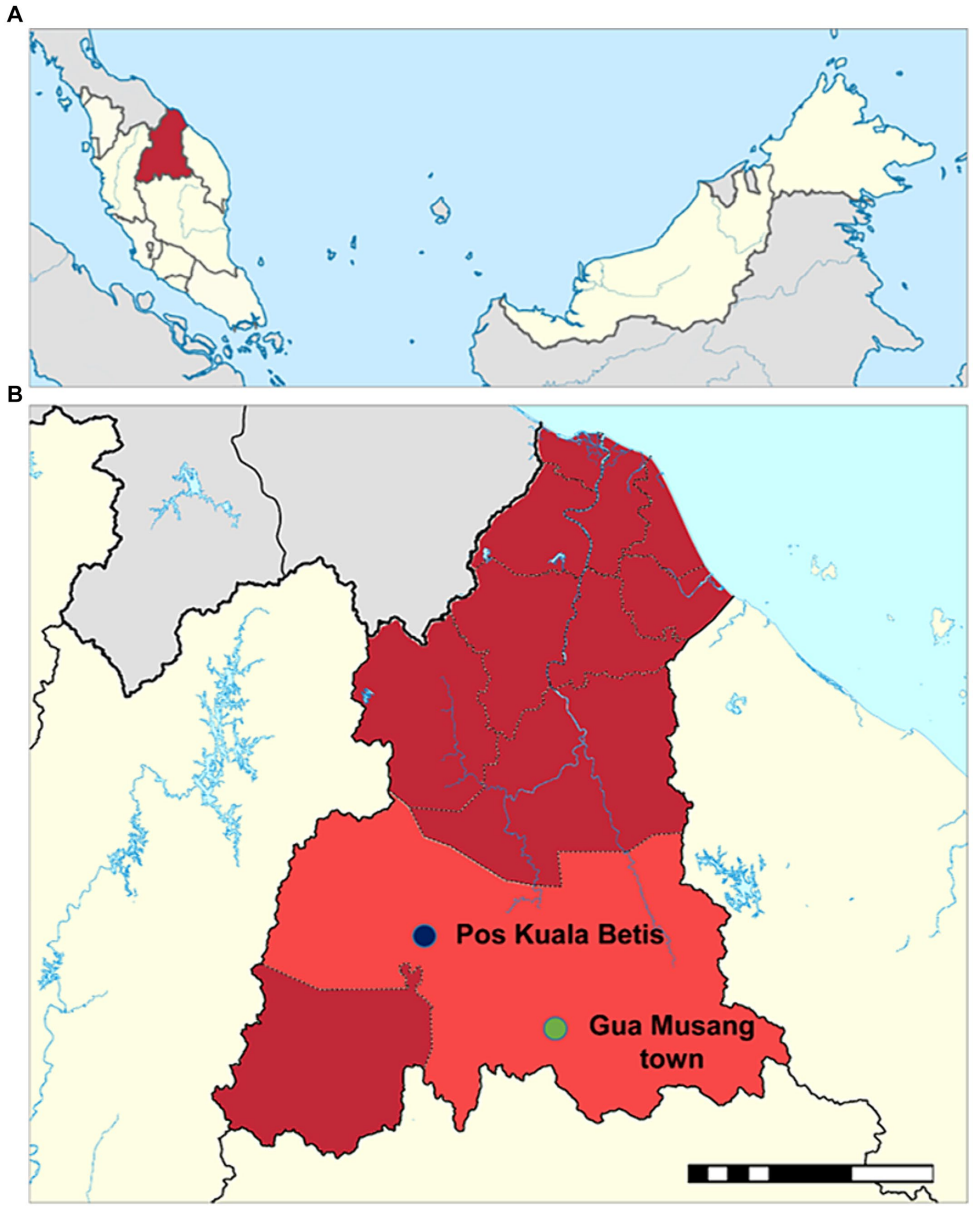
Map of the study setting. **(A)** Map of Malaysia showing the location of Kelantan State (dark red). **(B)** Map of Gua Musang district (light red) in Kelantan State showing the location of Pos Kuala Betis. The study in Pos Kuala Betis is located approximately 40 km from Gua Musang town.

The study was conducted in Pos Kuala Betis (latitude 4^o^53´22”N; longitude 101^o^45´30″E), a clustered rural resettlement of five villages (i.e., Angkek, Betak, Galas, Lambok and Podek) located at the Gua Musang district, Kelantan State, Peninsular Malaysia (Figure 1). The Temiar sub-ethnic of the Senoi was known to be the main indigenous OA in these villages. The present study is the first to assess the prevalence and severity of anaemia in the area and also builds on previous studies on malaria among these communities (25, 26). While the main economic activity was centred on agriculture, such as palm oil plantation, the livelihood of the villagers mainly depended on rubber-tapping, labourers, farmers and gathering and selling forest products (29).

### 2.3 Study design, sample size and sampling techniques

A community-based cross-sectional survey was carried out between June and July 2019 (29). All indigenous Temiar OA of both genders residing in the study villages and aged one and older were invited to take part in the study. The sample size for study participation was calculated using the following Cochran’s formula: *N* = z^2^p(1–p)/e^2^, where z is the confidence interval which is set at 95% (z-value of 1.96); (p) is the expected prevalence of anaemia of 68% from a previous study (23), and (e) is the allowed error margin which is set at 5%. In addition, contingencies were adjusted by adding another 30% of individuals, giving us a minimum of 435 participants to be sampled.

Participants were explained about the study protocol, and informed consent was documented. For the illiterate participant, informed consent was obtained in the presence of an independent literate witness. For children and adolescents below 18 years old, informed consent was obtained from parents or legal guardians. All participants were also informed of their right to withdraw from the study at any time without prior notice and prejudice. District healthcare providers and community leaders were purposely involved in the study to facilitate participation and cooperation among the community (29). Participants were divided into four groups following age stratification practices in Malaysia, namely infants and children (≤5 years), school-going children and adolescents (6–17 years), young adults (18–40 years) and older adults (>40 years). As a token of appreciation, the participants who contributed to the research were given some refreshments (e.g., snacks and drinks) at the end of the sampling activity.

### 2.4 Data collection

A structured questionnaire was used for collecting data that assessed factors associated with anaemia in the community. The questionnaire was developed from standard closed-ended questions adopted from a previous study by Munajat et al. (29). The questionnaire was written in English and then translated into Malay. A peripheral blood sample was obtained from each participant’s fingertip. The first and second droplets of blood were discarded after puncturing the side of the fingertip with a puncture needle, and blood was collected from the third drop. Hb level was measured with the HemoCue Hb 201 Analyzer (HemoCue, Sweden) and was expressed as g/L. Anaemia was classified as mild, moderate or severe based on the concentrations of Hb in the blood. According to WHO criteria (30), for children aged 6–59 months and pregnant women, anaemia is defined at Hb <110 g/L (100–109, 70–99, and < 70 g/L correspond to mild, moderate and severe anaemia, respectively). For children aged 5–11 years old, anaemia is defined as Hb <115 g/L, in which 110–114, 80–109, and < 80 g/L correspond to mild, moderate and severe anaemia, respectively. While for children 12–14 years old and non-pregnant women (15 years of age and above), anaemia is defined as Hb <120 g/L (110–119, 80–109, <80 g/L refers to mild, moderate and severe anaemia, respectively). Lastly, in men (15 years of age and above), anaemia is defined as Hb <130 g/L, in which 110–12.9, 80–109, <80 g/L correlate to mild, moderate and severe anaemia, reciprocally (30).

### 2.5 Statistical analysis

All survey data were double-entered into Microsoft Excel spreadsheets and cross-checked for errors. Data were processed and analysed using STATA/SE 13.1 statistical software package (StataCorp, United States). Differences in proportions were tested using the Chi-squared test or Fisher’s exact test. 95% confidence intervals (95% CI) were estimated to provide uncertainty surrounding the point estimates. Logistic regression analysis was used to identify factors associated with anaemia in the community. Gender, age group, and village were considered explanatory variables in the univariate analyses. The reference group for the logistic regression analysis was based on the lowest prevalence of anaemia in each category. All variables with a *p*-value of <0.50 from the likelihood ratio test in the univariate analyses were included in the multivariate logistic regression model, and stepwise backward elimination was used to identify the main risk factors for anaemia (31). A *p* < 0.05 was considered statistically significant.

## 3 Results

### 3.1 Characteristic of study participants

A total of 640 individuals from Pos Kuala Betis (representing 68% of the combined population in the five villages) participated in this study (Table 1). The surveillance coverage varied among villages from 50 to 90%, being mostly females (58%), and the age of participants ranged between 1 to 88 years old (mean [standard deviation; SD] age of 22.6 [17.8] years). A slight majority of the participants were school-age children and adolescents in the 6–17 age group (34.1%; 95% CI: 30.4–37.9). About 58% (95% CI: 54.1–61.8) of the participants reported enrolling in formal education at least once in their lifetime, with significant differences in education levels between the villages (*p* < 0.001). Most participants (44.4%; 95% CI: 39.7–49.2) were students with significant differences in each village (*p* = 0.028). The majority of employed participants (62.6%; 95% CI: 52.3–72.1) received less than Malaysian Ringgit (MYR) 500 (i.e., ≤USD105) per month. Of the entire participants, the majority 423 (86.7%; 95% CI: 83.3–89.6) had food security and significantly higher than those who experienced food insecurity between villages (*p* = 0.024).

**Table 1:** Demographic characteristic and anaemia status of indigenous Temiar Orang Asli population in five settlements of Pos Kuala Betis, Kelantan, Malaysia.

Characteristic	Overall	Angkek	Betak	Lambok	Podek	Galas	*p*-value^a^
Total population^b^, *N*	941	154	100	198	281	208	
Total number of respondents, *n* (%)	640 (68)	138 (89.6)	50 (50)	150 (75.8)	186 (66.2)	116 (55.8)	
Gender, *n* (%)							
Male	269 (42)	71 (51.5)	17 (34)	56 (37.3)	77 (41.4)	48 (42.4)	0.101
Female	371 (58)	67 (48.5)	33 (66)	94 (62.7)	109 (58.6)	68 (58.6)	
Age, mean (SD), years	22.6 ± 17.8	21.6 ± 16.8	24.9 ± 19.3	24.4 ± 19	20.5 ± 17.2	23.9 ± 17.8	0.206
Age group^c^, *n* (%), years							
≤5	104 (16.3)	27 (19.6)	8 (16)	18 (12)	35 (18.8)	16 (13.8)	0.607
6–17	218 (34.1)	43 (31.2)	17 (34)	53 (35.3)	68 (36.6)	37 (31.9)	
18–40	214 (33.3)	50 (36.2)	14 (28)	54 (36)	57 (30.6)	39 (33.6)	
>40	104 (16.3)	18 (13)	11 (22)	25 (16.7)	26 (14)	24 (20.7)	
Education level^d^							
No formal education	113 (23.4)	21 (21.2)	13 (33.3)	25 (20.7)	27 (19.9)	27 (30.3)	<0.001
Primary education	231 (47.7)	40 (40.4)	24 (61.5)	65 (53.7)	75 (55.1)	27 (30.3)	
Secondary or higher	140 (28.9)	38 (38.4)	2 (5.2)	31 (25.6)	34 (25)	35 (39.4)	
Occupation^d^							
Unemployed	57 (12.9)	13 (13.5)	1 (3.2)	16 (14.4)	13 (10.8)	14 (17.3)	0.028
Self-employed	76 (17.3)	28 (29.2)	3 (9.7)	14 (12.6)	19 (15.9)	12 (14.8)	
Students	195 (44.4)	39 (40.6)	16 (51.6)	45 (40.5)	58 (48.3)	37 (45.6)	
Housewives	88 (20.2)	10 (10.4)	8 (25.8)	32 (28.8)	25 (20.8)	13 (16.1)	
Government	23 (5.2)	6 (6.3)	3 (9.7)	4 (3.6)	5 (4.2)	5 (6.2)	
Monthly income^e^							
≤MYR500	62 (62.6)	22 (64.7)	3 (50)	12 (66.7)	16 (66.7)	9 (52.9)	0.836
>MYR500	37 (37.4)	12 (35.3)	3 (50)	6 (33.3)	8 (33.3)	8 (47.1)	
Food insecurity status^d^							
Food secure	423 (86.7)	89 (87.3)	38 (92.7)	110 (92.4)	108 (83.7)	78 (80.4)	0.024
Mild food secure	37 (7.6)	7 (6.9)	2 (4.9)	5 (4.2)	15 (11.6)	8 (8.3)	
Moderately food secure	22 (4.5)	5 (4.9)	1 (2.4)	4 (3.4)	6 (4.7)	6 (6.2)	
Severely food secure	6 (1.2)	1 (0.9)	0 (0)	0 (0)	0 (0)	5 (5.3)	
Hb level, mean (SD), g/dL	12.1 ± 2	12.2 ± 2	12.4 ± 1.3	12 ± 1.8	12 ± 2.3	12.3 ± 1.9	0.321
Anaemia^f^, *n* (%)							
No	354 (55.3)	78 (56.5)	35 (70)	72 (48)	102 (54.8)	67 (57.8)	0.092
Yes	286 (44.7)	60 (43.5)	15 (30)	78 (52)	84 (45.2)	49 (42.2)	

SD, standard deviation; MYR, Malaysian Ringgit.

a Comparison between village.

b Census from the Demographic Surveillance System of the District Health Office of Gua Musang.

c Based on the Department of Statistics Malaysia.

d Question asked for individuals ≥ 7 years.

e Question asked for employed individuals.

f Based on the WHO classification of anaemia based on blood haemoglobin level (30).

### 3.2 Anaemia-specific prevalence

Of all participants, the overall mean haemoglobin level and prevalence of anaemia were 12.1 ± 2 g/dL and 44.7% (95% CI: 40.8–48.6) and not significantly different between villages (*p* > 0.05) (Table 1). The anaemia-specific prevalence among participants in Pos Kuala Betis is shown in Table 2. The prevalence of mild, moderate and severe anaemia was 42.7% (95% CI: 36.9–48.6), 50.7% (95% CI: 44.7–56.6) and 6.6% (95% CI: 4.1–10.2), respectively. Although the prevalence of anaemia was highest among females (57%; 95% CI: 51.2–62.8), no significant difference was observed between the three anaemia severity classifications (*p* = 0.691). Nevertheless, the prevalence of anaemia varied significantly by age group (*p* < 0.001) and was highest in the ≤5 group for both moderate anaemia (43.4%; 95% CI: 35.2–51.9) and severe anaemia (42.1%; 95% CI: 20.3–66.5), followed by the 6–17 age group for mild anaemia (39.3%; 95% CI: 30.6–48.6). Albeit no significant differences were observed between anaemia severity classifications, a high prevalence of anaemic individuals was observed in those living in Podek village, with primary education, monthly income ≤MYR500 and food security with 29.4% (95% CI: 24.2–35.1), 45.7% (95% CI: 38.4–53.1), 62.5% (95% CI: 40.6–81.2) and 87.4% (95% CI: 81.7–91.9), respectively. Interestingly, the prevalence of anaemia was highest among students (53.9%; 95% CI: 46.1–61.7), with a significant difference observed between the three anaemia severity classifications (*p* = 0.002).

**Table 2:** Specific prevalence of anaemia among indigenous Temiar Orang Asli communities in Pos Kuala Betis, Kelantan, Malaysia.

	Anaemia	Mild	Moderate	Severe	*p*-value
Overall, *n* (%)	286 (100)	122 (42.7)	145 (50.7)	19 (6.6)	
Gender, *n* (%)					
Male	123 (43)	56 (45.9)	59 (40.7)	8 (42.1)	0.691
Female	163 (57)	66 (54.1)	86 (59.3)	11 (57.9)	
Age group, *n* (%), years					
≤5	74 (25.9)	3 (2.5)	63 (43.4)	8 (42.1)	<0.001
6–17	106 (37.1)	48 (39.3)	52 (35.9)	6 (31.6)	
18–40	66 (23.1)	44 (36.1)	20 (13.8)	2 (10.5)	
>40	40 (13.9)	27 (22.1)	10 (6.9)	3 (15.8)	
Village, *n* (%)					
Angkek	60 (21)	27 (22.1)	28 (19.3)	5 (26.3)	0.109
Betak	15 (5.2)	11 (9)	4 (2.8)	0 (0)	
Lambok	78 (27.3)	35 (28.7)	40 (27.6)	3 (15.8)	
Podek	84 (29.4)	27 (22.2)	48 (33.1)	9 (47.4)	
Galas	49 (17.1)	22 (18)	25 (17.2)	2 (10.5)	
Education level					
No formal education	43 (23.1)	29 (27.1)	11 (15.9)	3 (30)	0.069
Primary education	85 (45.7)	42 (39.3)	36 (52.2)	7 (70)	
Secondary or higher	58 (31.2)	36 (33.6)	22 (31.9)	0 (0)	
Occupation					
Unemployed	22 (13.3)	16 (17.2)	5 (7.8)	1 (12.5)	0.002
Self-employed	19 (11.5)	18 (19.4)	1 (1.6)	0 (0)	
Students	89 (53.9)	42 (45.2)	42 (65.6)	5 (12.5)	
Housewives	30 (18.2)	13 (13.9)	16 (25)	1 (12.5)	
Government	5 (3.1)	4 (4.3)	0 (0)	1 (12.5)	
Monthly income					
≤MYR500	15 (62.5)	14 (63.6)	1 (100)	0 (0)	0.320
>MYR500	9 (37.5)	8 (36.4)	0 (0)	1 (100)	
Food insecurity status					
Food secure	160 (87.4)	91 (83.5)	60 (92.3)	9 (100)	0.547
Mild food secure	12 (6.6)	9 (8.3)	3 (4.6)	0 (0)	
Moderately food secure	8 (4.4)	6 (5.4)	2 (3.1)	0 (0)	
Severely food secure	3 (1.6)	3 (2.8)	0 (0)	0 (0)	

MYR, Malaysian Ringgit.

### 3.3 Factor associated with anaemia

Results for all significant co-variables associated with anaemia in univariate analysis (*p* < 0.05) are provided in Table 3. These variables were further used to build multivariate models with stepwise forward selection. The final model in multivariate logistic regression showed that age groups of 6–17 (adjusted odds ratio [aOR] 0.38 [95% CI: 0.23–0.63], *p* < 0.001), 18–40 (aOR 0.18 [95% CI: 0.11–0.31], *p* < 0.001) and > 40 (aOR 0.25 [95% CI: 0.14–0.45], *p* < 0.001) were significantly associated with retaining the lower odds of anaemia in the population.

**Table 3:** Factors associated with anaemia among indigenous Temiar Orang Asli in Pos Kuala Betis, Kelantan, Malaysia.

Risk factor	Category	*N*	Anaemia rate, % (95% CI)	Crude OR (95% CI)	*p*-value	Adjusted OR (95% CI)	*p*-value
Gender	Male	269	45.7 (39.7–51.9)	1.00		1.00	
	Female	371	43.9 (38.8–49.2)	0.93 (0.68–1.28)	0.653	1.05 (0.69–1.58)	0.827
Age group	≤5	104	71.2 (61.4–79.6)	1.00		1.00	
	6–17	218	48.6 (41.8–55.5)	0.38 (0.23–0.63)	<0.001	0.38 (0.23–0.63)	<0.001
	18–40	214	30.8 (24.7–37.5)	0.18 (0.11–0.31)	<0.001	0.18 (0.11–0.31)	<0.001
	>40	104	38.5 (29.1–48.5)	0.25 (0.14–0.45)	<0.001	0.25 (0.14–0.45)	<0.001
Village	Angkek	138	43.5 (35.1–52.2)	1.00		1.00	
	Betak	50	30 (17.9–44.6)	0.55 (0.28–1.11)	0.098	0.78 (0.32–1.92)	0.592
	Lambok	150	52 (43.7–60.2)	1.41 (0.88–2.24)	0.149	1.57 (0.89–2.78)	0.123
	Podek	186	45.2 (37.9–52.6)	1.07 (0.69–1.67)	0.763	1.17 (0.66–2.06)	0.591
	Galas	116	42.2 (33.1–51.8)	0.95 (0.58–1.57)	0.843	1.02 (0.54–1.91)	0.957
Education level	No formal education	113	38.1 (29.1–47.7)	1.00		1.00	
	Primary education	231	36.8 (30.6–43.4)	0.95 (0.59–1.51)	0.821	0.47 (0.19–1.08)	0.078
	Secondary or higher	140	41.4 (33.2–50.1)	1.15 (0.69–1.91)	0.586	0.58 (0.24–1.39)	0.228
Occupation	Unemployed	57	38.6 (25.9–52.4)	1.00		1.00	
	Self-employed	76	25 (15.8–36.3)	0.53 (0.25–1.11)	0.095	0.53 (0.25–1.12)	0.095
	Students	195	45.6 (38.5–52.9)	1.33 (0.73–2.44)	0.347	1.25 (0.43–3.64)	0.683
	Housewives	88	34.1 (24.3–44.9)	0 82 (0.41–1.64)	0.581	0.82 (0.41–1.65)	0.569
	Government	23	21.7 (7.5–43.7)	0.44 (0.14–1.36)	0.155	0.43 (0.14–1.34)	0.155
Monthly income	≤MYR500	62	24.2 (14.2–36.7)	1.00		1.00	
	>MYR500	37	24.3 (11.7–41.2)	1.01 (0.38–2.60)	0.988	1.23 (0.33–4.53)	0.756
Food insecurity status	Food secure	423	37.8 (33.2–42.6)	1.00		1.00	
	Mild food secure	37	32.4 (18.1–49.8)	0.79 (0.39–1.61)	0.516	0.84 (0.39–1.81)	0.662
	Moderately food secure	22	36.4 (17.2–59.3)	0.94 (0.39–2.29)	0.890	1.17 (0.44–3.13)	0.750
	Severely food secure	6	50 (11.8–88.2)	1.64 (0.33–8.24)	0.546	1.34 (0.21–8.47)	0.758

CI, confident interval; MYR, Malaysian Ringgit; OR, odd ratio.

## 4 Discussion

This study determined the current status and risk factors of anaemia among the Temiar sub-ethnic indigenous OA in Peninsular Malaysia. Overall, this study has shown a high anaemia prevalence of 44.7%, particularly among children and adolescents and considered a severe level (i.e., >40%) based on the ranges of anaemia among indigenous populations globally (11). Although this figure was two folds higher than the anaemia incidence among the general Malaysian population, i.e., 24.2% (32), the prevalence of anaemia among the Temiar OA was within similar ranges reported in other indigenous tribes in Malaysia, i.e., the Senoi and Semai in Peninsular Malaysia (16, 17), and Penan and Iban in Malaysian Borneo (33, 34). However, these findings were contrary to a recent study conducted among the present-day hunter-gatherer of indigenous Negritos OA, with an overall anaemia prevalence of 68.4% (23). As compared to the other main tribes, the prevalence of glucose-6 phosphate dehydrogenase (G6PD) deficiency was reported to be high among Negritos OA (i.e., 46.6%); hence this could further risk them to have haemolytic anaemia (35). Apart from that, the drastic nutritional transition from a traditional to a new modern lifestyle, relocation to an unfavourable location and inability to cope with a new environment among Negritos communities also contributed to the high prevalence of anaemia (23). The fact that a high prevalence of anaemia among indigenous populations is widely distributed in Malaysia suggests that common disparities in health among these diverse groups of people are attributed to a higher risk of anaemia.

The finding of this study demonstrated that the prevalence of anaemia was significantly higher in children and adolescents (i.e., ≤17 years, 55.9%) compared to adults (i.e., ≥18 years, 33.3%). This is consistent with earlier local studies conducted among OA in Peninsular Malaysia (15, 17). The variation in prevalence and severity of anaemia tends to be convex with age, increasing in childhood and decreasing in adulthood (33). This may be because the child’s exposure to being anaemic rises as he or she gets older. It is common for children to be more active, curious, and eager to learn new things in their surroundings. However, many of them are still unaware of personal and appropriate hygiene practices and the consequences of exposing themselves to infectious organisms due to their early age. Meanwhile, as in agreement with a previous study by Muslim et al. (23), gender stratification in this study showed no difference between the prevalence of anaemia between females and males, although it is well acknowledged that females tend to be more anaemic as a result of physiological differences (7, 36).

The present study also revealed that anaemia-specific prevalence of both moderate and severe anaemia was highest in children aged less than five years old with 43.4 and 42%, respectively. Besides, the odds of anaemia were lower in the 6–17 group when compared to those in the ≤5 group in the population. These scenarios agree with previous reports (15, 23, 27), whereby the younger children from the indigenous population in Malaysia had a higher prevalence of anaemia than older children. In the OA community, older children are usually enrolled in boarding schools provided by the government. It is postulated that a feeding intervention implemented in the boarding school could reflect the lower prevalence of anaemia among older children. Previous studies have shown that the contributing factors of anaemia among children of the indigenous population in Malaysia are largely due to poor dietary intake of iron, recurrent infections and low socioeconomic status (27, 28). Furthermore, preschoolers depend on their caregivers to provide food for most of the day. Because of that, a caregiver with a lack of knowledge of proper nutrition, low socioeconomic status, unemployment and low level of education attributed to childhood malnutrition. This issue deserves attention because chronic anaemia during childhood has been shown to be associated with retardation in physical development and cognition, while severe anaemia is responsible for more than half of the deaths in children under 5 years of age (37).

Local variation in anaemia prevalence was also observed among communities in the same clustered rural settlement. For example, the present study showed that anaemia prevalence in the five villages in Pos Kuala Betis ranged from 30 to 52%. The heterogeneity in anaemia prevalence within indigenous communities due to the difference in location and access to food has also been reported in other settings (38). Like many other indigenous communities globally (11), OA communities in Malaysia are often located in deeply remote areas with a lack of basic amenities such as piped water supply, electricity, toilet facility, garbage disposal service, as well as limited access to government health programs (21, 39). Tackling these issues could ultimately reduce intestinal parasitic transmission, subsequently lowering the prevalence of anaemia in OA communities.

A number of caveats should be considered in this study. First, this study lacks of potential sample representative. The participants were drawn from a single remote settlement, which may not fully capture the diversity and variability of the broader Temiar OA communities in Peninsular Malaysia. This limitation poses a challenge to the generalizability of our findings across the entire Temiar population. Furthermore, the reliance on a single settlement limits our ability to account for intra-community variations within the Temiar population. The sample may not encompass the full spectrum of age, gender, and socio-economic status distributions present in the wider community, potentially leading to biased estimates of anaemia prevalence and severity. To address these limitations in future research, it would be beneficial to include participants from multiple settlements across different regions. Second, although the cross-sectional nature of the research design was efficient and cost-effective, it has an inherent selection bias and does not allow causality to be established. While the cross-sectional approach allows for the identification of associations and prevalence at a specific point in time, it does not provide insights into the temporal sequence of events or the direction of these associations. This limitation is particularly important when considering the multifactorial nature of anaemia, where various factors such as infections, and socio-economic conditions may interplay. To gain a more comprehensive understanding of the factors contributing to anaemia in the Temiar population, longitudinal studies are essential. Third, the lack of data on micronutrient deficiencies, infectious diseases and genetic predispositions is important for understanding the causes of anaemia in the study population. While the study provides valuable data on the prevalence and severity of anaemia, it does not delve into the potential contributors to this condition. This omission hinders our ability to identify and address the specific causes of anaemia in this community. Micronutrient deficiencies, particularly of iron, vitamin B12, and folate, are well-known contributors to anaemia (5, 6). Without assessing the nutritional status of the participants, it is challenging to determine the extent to which these deficiencies may be driving the observed anaemia prevalence. Similarly, infectious diseases, including helminth infections, and chronic inflammatory conditions, can significantly impact haemoglobin levels and anaemia risk. The absence of data on the presence or prevalence of these infections in the study population limits our understanding of their role in anaemia aetiology. Fourth, the use of capillary blood instead of venous samples can also constitute a source of bias. At the moment when the surveyor pricks the skin and collects blood drops, the Hb can be diluted with extracellular fluid through manipulation of the subject’s finger (40). The choice of capillary sampling was primarily driven by practical considerations, including the ease of collection in remote field settings and the reduced discomfort for participants. However, it is important to acknowledge that this choice may limit the comparability of our results with studies that utilize venous blood samples. Nonetheless, this technique offers many practical advantages and does not affect the quality of diagnosis at the population level (41).

Despite these limitations, studying anaemia prevalence among the Temiar OA community is crucial for several reasons. This study highlights the health disparities faced by indigenous populations, who often have limited access to healthcare and nutritional resources. Understanding anaemia prevalence in this specific community can help prioritize public health efforts and allocate resources effectively to address their unique health challenges. Additionally, data from the present study provide a baseline for monitoring health trends over time and assessing the effectiveness of interventions aimed at improving nutritional and health outcomes. This research also raises awareness about the broader social determinants of health affecting the Temiar OA community, such as poverty, education, and food security, which are critical for developing comprehensive health policies. Ultimately, these studies are a vital step towards achieving health equity and improving the overall well-being not just of the Temiar OA community, but of all indigenous ethnic groups in the country.

In conclusion, anaemia constitutes a major health problem, particularly among the school-age children of the Temiar sub-ethnic indigenous OA communities in Peninsular Malaysia. The magnitude of anaemia in this study carries public health importance, particularly in planning programs for community health and could help develop intervention strategies and target high-risk subpopulations in this vulnerable population group. Furthermore, the high prevalence of anaemia among the Temiar OA community underscores the urgent need for targeted interventions that address potential nutritional deficiencies, improve healthcare access, and tackle underlying socioeconomic determinants of health. By implementing culturally sensitive strategies and engaging community stakeholders, public health efforts can effectively reduce anaemia prevalence and improve overall health outcomes among the indigenous communities. While the study provides valuable insights into anaemia prevalence and severity among the Temiar OA in a specific settlement, caution should be exercised when extrapolating these findings to the broader population. A more comprehensive approach involving multiple settlements and diverse demographic groups would enhance the robustness and generalizability of future research in this area. Further research also needs to be done to ascertain the exact cause of anaemia in this community.

## Data availability statement

The original contributions presented in the study are included in the article/Supplementary material, further inquiries can be directed to the corresponding author.

## Ethics statement

The studies involving humans were approved by Medical Ethics Committee of the National University of Malaysia. The studies were conducted in accordance with the local legislation and institutional requirements. Written informed consent for participation in this study was provided by the participants' legal guardians/next of kin.

## Author contributions

ZI: Conceptualization, Data curation, Formal analysis, Funding acquisition, Investigation, Methodology, Project administration, Resources, Software, Supervision, Validation, Visualization, Writing – original draft, Writing – review & editing. WW: Investigation, Methodology, Writing – review & editing. MS: Investigation, Methodology, Writing – review & editing. NG: Investigation, Methodology, Writing – review & editing. NH: Investigation, Methodology, Writing – review & editing. SA: Investigation, Methodology, Project administration, Writing – review & editing. AM: Investigation, Methodology, Writing – review & editing. SC: Conceptualization, Investigation, Methodology, Validation, Writing – review & editing. MM: Conceptualization, Investigation, Methodology, Resources, Validation, Writing – review & editing.
